# Review of femoroacetabular impingement syndrome

**DOI:** 10.1093/jhps/hnae034

**Published:** 2024-10-15

**Authors:** Fernando Gómez-Verdejo, Elsa Alvarado-Solorio, Carlos Suarez-Ahedo

**Affiliations:** Trauma Department, Instituto Nacional de Rehabilitación Luis Guillermo Ibarra, Calzada México-Xochimilco No. 289 Colonia Arenal de Guadalupe Delegación, Tlalpan C.P., Ciudad de México 14389, México; Rehabilitation Department, Instituto Nacional de Rehabilitación Luis Guillermo Ibarra, Calzada México-Xochimilco No. 289 Colonia Arenal de Guadalupe Delegación, Tlalpan C.P., Ciudad de México 14389, México; Hip and Knee Adult Reconstruction Department, Instituto Nacional de Rehabilitación Luis Guillermo Ibarra, Calzada México-Xochimilco No. 289 Colonia Arenal de Guadalupe Delegación, Tlalpan C.P., Ciudad de México 14389, México

## Abstract

Femoroacetabular impingement syndrome (FAIS) is a common condition of the hip that can cause significant damage to the joint, leading to degeneration and osteoarthritis. FAIS constitutes an abnormal and dynamic contact between the femoral head–neck junction and the acetabular rim, resulting from altered bone morphology at one or both sites. Repetitive trauma at the site of impingement generates progressive damage to the acetabular labrum, chondrolabral junction, and articular cartilage. Proper diagnosis based on patient symptoms, specific clinical signs, and imaging findings will guide treatment and ultimately allow preservation of the native hip joint. Common symptoms in patients with FAIS include pain, clicking, catching, buckling, stiffness, giving way, and a limited range of motion of the hip. Specific clinical maneuvers can aid diagnosis, such as flexion adduction internal rotation and flexion abduction external rotation tests. Imaging diagnosis includes orthogonal hip and pelvis X-ray views, as well as magnetic resonance imaging/magnetic resonance arthrogram imaging. Initial treatment of FAIS can be conservative and include physical therapy, intra-articular injections, and activity modification. Currently, the preferred surgical management consists of hip arthroscopy, which aims to correct bony abnormalities, repair or reconstruct labral lesions and address other intra-articular and extra-articular derangements as needed.

## Introduction

Femoroacetabular impingement syndrome (FAIS) is a common condition of the hip that can cause significant damage to the joint, leading to degeneration and osteoarthritis (OA). Proper diagnosis based on patient symptoms, specific clinical signs, and imaging findings will guide treatment and ultimately allow preservation of the native hip joint. Our review aims to provide a comprehensive overview of the clinical signs, symptoms, and imaging findings associated with femoroacetabular impingement (FAI), as well as the preferred current surgical treatment, hip arthroscopy, highlighting the essential topics for proper diagnosis and treatment of this prevalent condition, especially in young patients. A literature search was conducted utilizing PubMed, EMBASE, and the Cochrane Library aiming to identify peer-reviewed, English language studies in order to analyze current practices regarding diagnosis and treatment of FAIS.

In addition to the database search, references in the selected articles were evaluated for additional relevant articles.

## Definition of FAIS

FAI constitutes an abnormal contact between the femoral head–neck junction and the acetabular rim, resulting from altered bone morphology at one or both sites. Repetitive trauma at the site of impingement generates progressive damage to the acetabular labrum, chondrolabral junction, and articular cartilage.

This abnormal contact tends to occur with specific movements or positions of the joint; hence, it is considered a dynamic and complex hip derangement.

To standardize definitions and diagnosis criteria regarding FAI, the 2016 Warwick Agreement on FAI decided that the term “femoroacetabular impingement syndrome” should be preferred over other currently usually employed terms such as “asymptomatic FAI” or “radiological FAI” [1].

Cam and pincer “morphology” is the currently preferred terminology for the description of bony abnormalities, as opposed to the previously employed terms of cam and pincer “lesions” [1].

### Pincer vs. cam vs. mixed types

Three morphological variants of FAI exist: pincer, cam, and mixed [2, 3]. Although extra-articular impingement entities such as central iliopsoas impingement, subspine impingement, ischiofemoral impingement, and greater trochanteric-pelvic impingement have been described and should be known by the clinician, their detailed description falls outside the scope of this review [4].

#### Pincer

**Figure 1.**
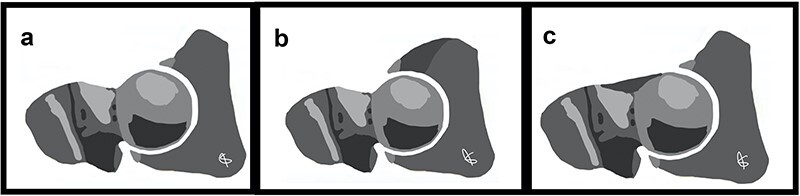
Schematic representation of hip: (a) normal hip, (b) hip with pincer-type impingement (the dark gray zone represents the acetabular over-coverage), and (c) hip with cam-type impingement (the dark gray zone represents the femoral head–neck junction abutment).

Pincer morphology refers to an over-coverage of the femoral head by the acetabulum. This over-coverage may be global (e.g. coxa profunda and protrusio acetabuli), or focal, located in the anterolateral region of the acetabular rim (e.g. acetabular retroversion and subspine impingement) [5]. Regarding acetabular retroversion as a cause for pincer morphology, recent studies suggest that posterior underdevelopment of the acetabulum might be the cause, rather than anterior overgrowth [6, 7] (Fig. 1b).

#### Cam

Cam refers to an eccentric part added to a rotating device [8], such as a camshaft in an internal combustion engine, which converts rotatory motion into a reciprocating motion for engine valve opening and closure. Similarly, the femoral head rotational movement within the acetabulum is converted into a linear displacement secondary to the impingement of the eccentric bone prominence found in the femoral head–neck junction against the acetabular rim (Fig. 1c). This linear displacement results in compression and shear forces with subsequent labral tears and articular cartilage delamination at the anterolateral portion of the acetabulum, the most common site of impingement.

This abnormal motion results in progressive damage to the acetabulum’s chondrolabral junction, leading to labral tears and articular cartilage delamination at the anterolateral portion of the acetabulum. Cam morphology tends to be more common in male patients [7]. A systematic review and meta-analysis by Nepple *et al*. [9] reported that male sport-specific high-level athletes have up to 8.0 times the risk of developing a cam morphology vs. controls. The sports that were found to have greater risk were hockey, basketball, and possibly soccer when performed at a high level during adolescence [9].

#### Mixed

Mixed morphology refers to the coexistence of a cam and pincer in the same hip.

Of relevance, the abnormal displacement generated by FAIS may also result in chondral damage at the posteroinferior region of the acetabulum due to repetitive subluxation of the femoral head, referred to as “contrecoup” lesions, and these lesions may occur in the presence of isolated cam or mixed morphologies [10].

In 2013, Clohisy *et al*. reported on a multicenter, prospective, longitudinal cohort of 1076 patients (1130 hips) and found that 47.6% of the patients had cam morphology, 44.5% had a mixed (cam/pincer) morphology, and 7.9% had pincer morphology [11]. This study indicates that the most prevalent morphology is cam, while previously, there had been reports of mixed morphology being the predominant [12].

## Risk factors

The development of FAI is thought to be influenced by cumulative and repetitive mechanical overloads at the hip joint. A relationship between occupational exposure to such loads and FAI development remains unclear in potential high-risk occupational groups (e.g. military personnel and athletes). A high prevalence of FAI morphology in high-level athletes has been previously identified [13, 14] According to the systematic review by Canetti *et al*. FAI is more likely to occur in occupations involving high-intensity and high-impact physical activities, as well as those with repetitive and supraphysiologic hip loading. Unfortunately, the studies in this review did not detail which specific occupational tasks, and at which frequencies and durations, would increase the likelihood of FAI.

## Influence of pelvic sagittal balance

FAI is a dynamic derangement of the hip; with this in mind, the relationship between spinal mobility, pelvic incidence, hip mobility, and other spinopelvic parameters has been investigated. Diminished spine flexion with normal or greater hip flexion has been documented to be present in symptomatic FAI patients. Reduced spine flexion is compensated with increased pelvic tilt and hip flexion when sitting, which, in turn, may generate or increase impingement [15].

## Bilaterality

According to a recent study by Hale *et al*. [16], the incidence of FAI has significantly increased over the past few years. With a rate of 54.4 diagnoses per 100 000 person-years, the diagnosis of FAI has been on a steady rise since the year 2000 and continues to do so. Although most patients present with symptoms in only one hip, bilateral FAI can also occur. An analysis of 641 patients undergoing surgery for FAI, reporting a 21% incidence of bilaterally symptomatic FAI [15]. Furthermore, many patients have radiographic signs and findings indicative of FAI even when they do not exhibit symptoms [17]. Radiographic evidence of FAI was found in 78% of the contralateral hips in patients with a painful hip; only 26% had bilateral symptoms, according to Allen *et al*. [18].

## Clinical diagnosis

The 2016 Warwick Agreement on FAI syndrome established that diagnosis should be made based on a triad consisting of symptoms, clinical signs, and imaging findings [1].

### Symptoms

Common symptoms in patients with FAI syndrome include pain, clicking, catching, buckling, stiffness, giving way, and a limited range of motion. Pain is the most common symptom, usually has a mechanical presentation, and tends to be found in the inguinal region. Nevertheless, pain location has been variable, with some patients describing pain in the lateral region of the hip, the buttock, the thigh, or even the lower back or knee [17]. In this respect, an intra-articular local anesthetic injection may aid the diagnosis when there may be doubt regarding the intra-articular origin of the pain [19].

Gait pattern, muscle weakness, and tenderness around the hip should be examined and noted.

The passive range of motion should be compared to the contralateral side. Trendelenburg gait will usually indicate hip abductor weakness or lesion, while in-toeing or out-toeing in the context of suspicion of FAIS may indicate abnormal femoral anteversion [20]. Tenderness about the hip may indicate FAIS if it is located in the groin area, while tenderness over the greater trochanter or posterior hip may point to pathology of the proximal iliotibial band, sacroiliac joint, or other types of extra-articular impingement.

### Signs and specific physical examination maneuvers

The flexion adduction internal rotation is the most widely used clinical maneuver to aid in diagnosing FAIS; it is a highly sensitive (80%) but not a specific (24%) test [21–23]. The test is performed with the patient in the supine position; the clinician flexes the hip to 90°, and then adduction and internal rotation of the femur is performed. The test is considered positive if the patient reports pain or clicking.

The flexion abduction external rotation test is also employed to aid FAIS diagnosis. It is performed with the patient in the supine position; the examiner flexes, abducts, and rotates the hip and places the ipsilateral foot above the contralateral knee. The examiner then applies a slight downward force on the thigh to counter any guarding while ensuring that the patient’s pelvis does not tilt. The distance from the lateral epicondyle to the examination table is measured, and comparison is performed with the unaffected side. This test’s diminished external rotation range signifies anterior capsular tightness and iliopsoas irritation. Anterior pain elicited during this test indicates intra-articular pathology, and posterior pain can indicate posterior hip pathology or sacroiliac joint pathology. Sensitivity of this test has been reported to range from 41% to 97% and specificity from 18% to 100% [20, 23].

The internal rotation over pressure test is performed with the patient in the supine position; the examiner then flexes the hip and applies internal rotation while maintaining axial compression through the limb. The test is considered positive if it pain is elicited, indicating labral pathology. Sensitivity for this test has been reported to be 75%–89% [23, 24].

The scour test or labral stress test is performed with the patient in the supine position. Starting with the affected leg in flexion and internal rotation, axial compression is applied while simultaneously abducting and extending the hip. Pain with this maneuver indicates anterior labral pathology. Sensitivity has been reported to range from 50% to 100% [23, 24].

## Imaging diagnosis

### Radiology (projections and measurements)

Initial imaging diagnosis of hip impingement must include orthogonal views of the acetabulum and femoral head. This may be accomplished with several standard views, including an antero-posterior (AP) pelvis and Dunn 45°. Additional views may include false-profile, cross-table lateral, Dunn 90°, and frog-leg lateral views. Dunn 45° is preferred over frog leg, and Dunn 90° is preferred for cam morphology identification [25, 26].

The clinician should assess each projection. The quality of the images and proper acquisition must be verified first. After confirmation of adequate image quality, several specific measurements and signs must be deliberately investigated and noted:

#### AP pelvis

AP pelvis should be evaluated for hip joint space initially. This is an important parameter, as an articular space of 2 mm or less has been correlated with advanced OA and poor clinical results with hip arthroscopy. Hence, alternative interventions are advised in the presence of these findings [25]. Articular space should be measured at three points: the medial, central, and lateral edges of the acetabular sourcil. Measurements should be made at 90° from the subchondral bone [2].

Some of the most relevant measurements of acetabular morphology to be assessed on the AP pelvis view include Wiberg’s lateral center-edge angle and Tönnis angle.

The femoral alpha angle should be measured in the AP pelvis as well. However, measuring this angle on the Dunn 45° view is more critical as cam morphology is predominant in the anterosuperior femoral head–neck junction [26].

**Figure 2.**
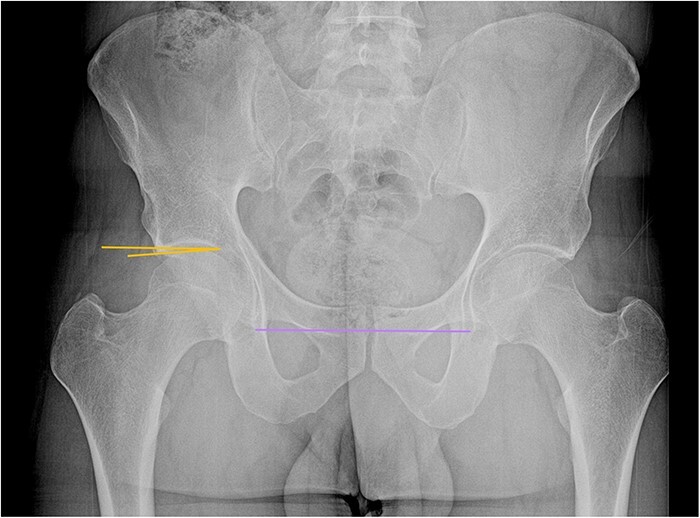
AP pelvis of a male patient with a right Tönnis angle of −5°, indicating pincer morphology. Note the line marked between the lower borders of both teardrops, used as a horizontal reference.

The Tönnis angle is measured between a horizontal line (typically derived from a line connecting both radiological Köhler’s teardrops) starting at the medial border of the acetabular sourcil and a second line extending from the medial to the lateral edges of the acetabular sourcil (Fig. 2) [2]. Reference values for the Tönnis angle are <0° for pincer morphology, 0–10° for normal, and >10° hip dysplasia [2].

**Figure 3.**
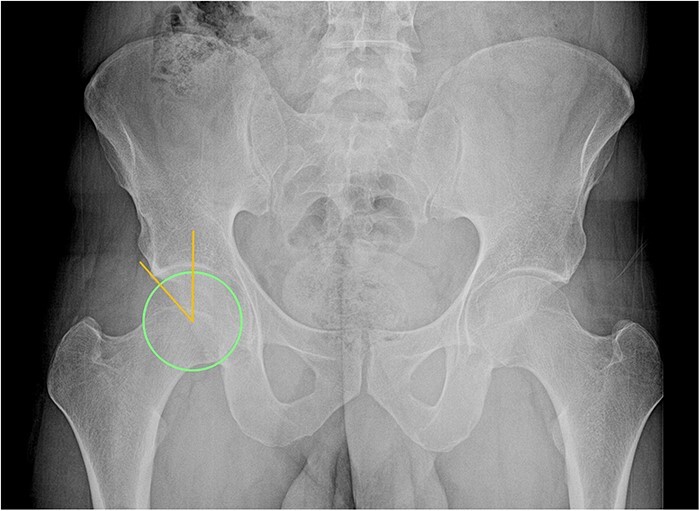
AP pelvis of a male patient with a right Wiberg’s center-edge angle of 42°, indicating pincer morphology.

The lateral center-edge angle of Wiberg is measured between a vertical line that extends upward from the center of the femoral head and a line that extends from the center of the femoral head to the lateral edge of the acetabular rim or sourcil (Fig. 3). Reference values for this angle are <20° for hip dysplasia, 20–25° for borderline dysplasia, 26–40° for normal and >40° for pincer morphology [2].

#### Dunn 45°

**Figure 4.**
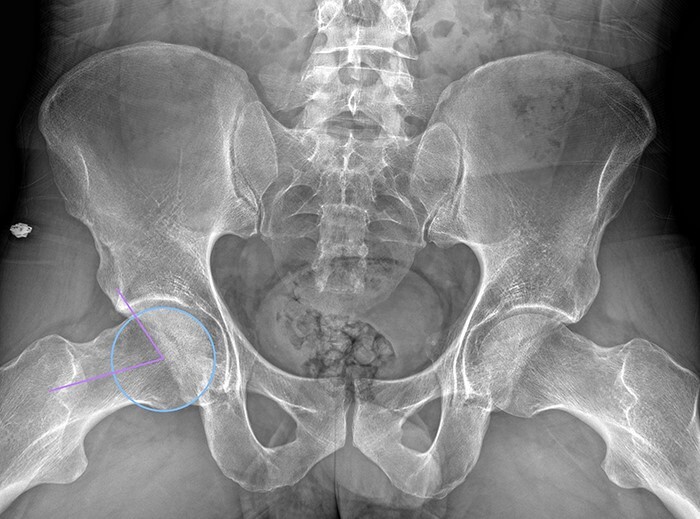
Dunn 45° view of a male patient with a right alpha angle of 75°, indicating cam morphology.

Dunn 45° view is the preferred method of assessment of the asphericity of the femoral head, as it is the most sensitive imaging study for evaluation of cam morphology [26]. Femoral head asphericity is quantified with the alpha angle; this measurement is made between a line drawn from the center of the femoral head and extending through the center of the femoral neck and a second line extending from the center of the femoral head to the point where the anterior portion of the femoral head loses its sphericity. To determine this last point, a perfect circle tool is employed (Fig. 4). Alpha angle cut-off is debated, with reported cut-offs ranging from >50° to >60° as abnormal [2, 27, 28].

### Magnetic resonance imaging/magnetic resonance arthrogram (common findings and normal anatomic variants)

**Figure 5.**
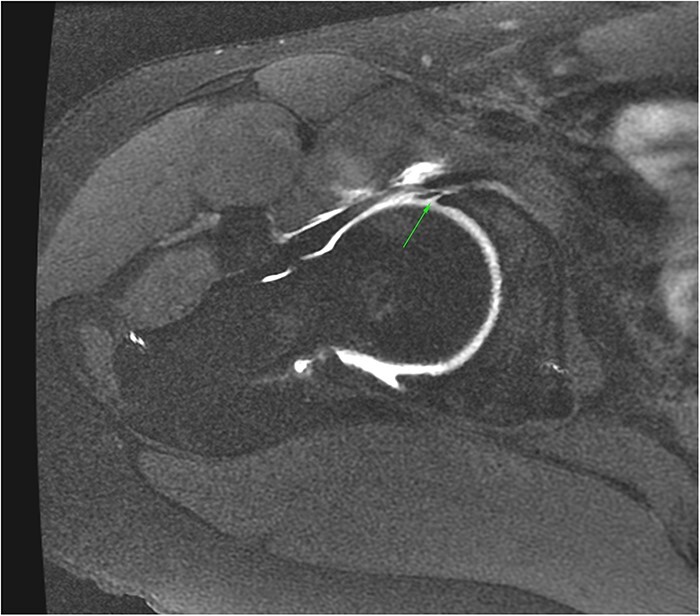
Axial MRA image of a right hip demonstrating a labral tear (arrow).

Magnetic resonance imaging (MRI) and magnetic resonance arthrogram (MRA) imaging are the preferred cross-sectional imaging modalities for evaluating FAIS. Although computed tomography has been recommended as a helpful tool for 3D imaging of cam and pincer morphology, MRI/MRA combined with previously described X-ray projections can yield sufficient information for clinical decision-making in most cases [26, 27]. MRI is useful for identifying different intra-articular pathology of the hip related to FAIS. At the same time, MRA is preferred for identifying intra-articular derangements commonly encountered in FAIS, such as labral tears, paralabral and impingement cysts, cartilage delamination, and bone edema (Fig. 5). It should be noted that although MRA has been considered a better option for visualization of intra-articular pathology, most comparative studies between MRI and MRA of the hip have been performed in 1.5T equipment, and more modern 3 T tesla resonators may yield images comparable to 1.5T MRA [27, 29, 30].

The main structures one should examine in MRI/MRA of the hip in the context of FAIS are the labrum, cartilage, osseous morphology, and subchondral bone/marrow [27].

Other structures that must be evaluated are the ligamentum teres, capsule, psoas tendon, gluteus medius, minimus tendon, hamstring tendons, and rectus femoris tendon [27].

## Treatment

Treatment of FAIS may be approached conservatively or surgically depending on several factors including patient characteristics such as age, functional status, labral and cartilage integrity, and symptom severity.

### Nonsurgical management

Nonsurgical management of FAI can include basic physical therapy options such as stretching, strengthening exercises, and manual therapy. Physical therapy improves hip flexibility, strength, and overall function and has been reported to be successful in up to 70% of patients [31, 32]. The addition of trunk and core stabilization exercises in conservative treatment has been shown to improve clinical outcomes by improving spinopelvic mechanics, which may emphasize the dynamic nature of FAI [33]. In some cases, the use of corticosteroid injections, platelet-rich plasma, or hyaluronic acid injections can also be considered. However, the results of these treatments can vary, and more research is needed to evaluate their efficacy. It is relevant to note that despite conservative treatment being accepted as the first option for most patients, conversion to arthroscopy has been reported in up to 70% of patients [34]. In conclusion, nonsurgical management is often the first line of treatment for FAI, but surgical management may be considered when it fails to improve symptoms.

### Surgical management

**Figure 6.**
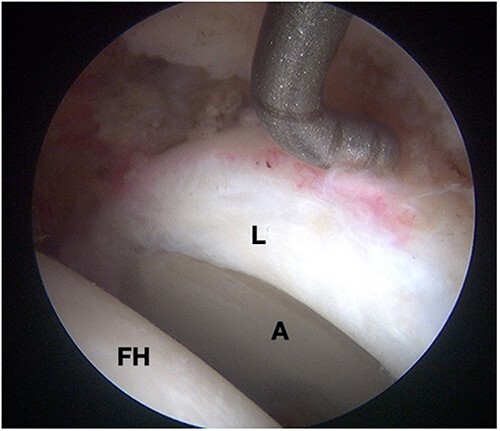
Intraoperative photographs (hip arthroscopy) of a left hip under traction (L, labrum; A, acetabulum; FH, femoral head).

Surgical management of FAI can be accomplished by open or arthroscopic techniques. Open techniques include surgical femoral head dislocation, periacetabular osteotomy, and femoral derotational osteotomy. Arthroscopic management of FAI has gained popularity in recent years. Though technically challenging, it has proved to provide faster recovery, return to sports, and fewer complications when compared to open alternatives (Fig. 6) [32, 35]. Nevertheless, open techniques may still be preferred over arthroscopic surgery in instances such as coxa profunda, global acetabular retroversion, and proximal femoral deformities [32, 36].

Indications for arthroscopic surgical management of FAI include previous failed conservative treatment, symptom improvement after intra-articular injection (indicating intra-articular pathology), and alpha angle >65° [32, 37]. Contraindications include an articular space of <2 mm, ≥50% joint space narrowing, obesity that precludes access to the joint, and arthritic changes of Tönnis Grade ≥2 [32, 37].

Arthroscopic treatment of FAI has been reported to yield significant improvements in pain and function evaluated through patient-reported outcome scores with a low complication rate and low reoperation rate [38]. Additionally, results of arthroscopic surgical management for FAI have been reported to outperform conservative treatment [39].

Minimally invasive approach to managing FAI through arthroscopy aims to correct hip morphology, acetabular offset, and coverage through acetabular rim and femoral cam resection when either or both components are present, while also addressing chondral and labral pathology, mainly through labral repair or reconstruction [25]. Anteriorly labral resection was more widely performed, but it has now been demonstrated that preserving the chondrolabral junction and re-establishing the labral suction seal yield better clinical results and better preserve the hip function [40–42]. The aim of rim and/or cam resection is to eliminate the mechanical cause of hip pathology, as restoring the labrum without modifying the bony anatomy will inevitably lead to continued symptoms and retear.

A recent study by Kucharik *et al*., which compared labral repair vs. debridement, included 204 hips and found a 5% conversion rate to total hip arthroplasty at 10 years for the labral repair group, contrasting with 21.9% for the labral debridement group [42]. In the same study, OA and older age were identified as risk factors for conversion.

Outcomes after arthroscopic hip surgery for FAI have been reported to be satisfactory. In a recent systematic review focused on mid- to long-term outcomes of hip arthroscopy where 13 studies including 1571 hips were analyzed, an increase in the modified Harris Hip Score from 52–63.5 preoperatively to 80.2–87.4 postoperatively was observed. In the same systematic review, an increase in the Hip Outcome Score–Sports-Specific Subscale from 37.3–51.2 preoperatively to 74.5–85.7 postoperatively was reported [43].

## Postsurgical management

Current rehabilitation protocols after hip arthroscopy break up the process into four main phases, yet there are multiple variations. The most frequently modified factors are the time assigned to each phase and the time from surgery to weight-bearing. Based on the review published in 2021 by Bistolfi *et al*. [44], the primary goals and exercises in each phase are discussed in the next subsections.

### Phase I

Phase I includes pain control, maximum joint protection (weight-bearing restriction), and strength and mobility maintenance (isometric and functional exercises).

### Phase II

Phase II includes restoration of range of motion, muscular re-education, functional strength, and gait progression (functional, targeted exercises, and cardiovascular fitness, e.g. cycling, swimming, and aquatic therapy).

### Phase III

Phase III includes gait re-education, strength optimization, and return to preinjury condition (recreational activities) through targeted exercises (hip flexors and core), plyometrics, and sport-specific drills.

### Phase IV

All restrictions are lifted. Mobility should be equal to or better than presurgical. Emphasis is made on muscular strength: weight machines, spinning, etc., and sport-specific drills.

Progression of rehabilitation should always consider the patient’s age, injury type, preinjury activity level, and postsurgical pain.

## Summary

FAI is a condition that occurs when there is an abnormal contact between the femoral head–neck junction and the acetabular rim in the hip joint. FAI has three morphological variants: cam, pincer, and mixed. The cause of FAI is influenced by physical activity and may have a genetic component. Clinical diagnosis of FAI is based on symptoms, clinical signs, and imaging results. Treatment options include physical therapy, injections, and surgery. Postsurgical rehabilitation should be tailored to the patient’s age, injury type, and activity level.

## Data Availability

No new data were generated or analyzed in support of this research.
